# A 6-week ketogenic diet in treatment-resistant depression: a prospective single-arm feasibility pilot trial

**DOI:** 10.3389/fnut.2026.1870412

**Published:** 2026-07-22

**Authors:** Cemre Cukaci, Ulrich Rabl, Richard Frey

**Affiliations:** 1 Department of Psychiatry and Psychotherapy, Medical University of Vienna, Vienna, Austria; 2 Comprehensive Center for Clinical Neurosciences and Mental Health, Medical University of Vienna, Vienna, Austria; 3 Department of Psychiatry and Psychotherapeutic Medicine, University Hospital St. Pölten, Karl Landsteiner University of Health Sciences, Krems an der Donau, Austria

**Keywords:** mood disorder, gut-brain axis, ketogenic diet, ketogenic metabolic therapy, metabolic psychiatry, treatment-resistant depression

## Abstract

**Background:**

Prior evidence for the ketogenic diet (KD) interventions in treatment-resistant depression (TRD) was very limited when recruitment for the present pilot trial began on 1 November 2023. This pilot trial aimed to examine feasibility, adherence, safety, and preliminary clinical signals in patients with TRD treated with a 6-week KD intervention.

**Methods:**

This prospective, single-arm, open-label pilot trial was conducted in adult patients with TRD who had a baseline Montgomery-Åsberg Depression Rating Scale (MADRS) score of at least 20. The intervention consisted of a 6-week KD; preexisting psychopharmacological treatment had to be stable before study entry and was not changed during the intervention. Adherence was monitored by daily fasting capillary *β*-hydroxybutyrate measurements. The MADRS was assessed at baseline and after 2, 4, and 6 weeks in all participants. A response was defined as a ≥ 50% reduction in the MADRS score from baseline; remission was defined as a MADRS score of ≤10.

**Results:**

A total of 13 eligible patients were offered study participation; 5 declined, and 8 were enrolled. Four patients completed the full 6-week intervention. One patient discontinued after week 2, one patient was excluded after a revised diagnosis, and two patients discontinued because nutritional ketosis was not achieved. Nutritional ketosis was documented in all completers and was accompanied by marked symptomatic improvement in three of the four completers. Among the completers, two patients met the remission criteria at week 6, whereas one showed partial improvement and one showed no relevant antidepressant benefit despite sustained nutritional ketosis. The intervention was generally feasible, but recruitment and retention were challenging.

**Conclusion:**

In this small prospective pilot trial, a KD was feasible in a subset of patients with TRD and was associated with improvement in depressive symptoms in three of the four completers. However, recruitment difficulties, early discontinuations, and heterogeneity of clinical responses highlight the need for larger controlled studies.

## 1 Introduction

Treatment-resistant depression (TRD) remains a major clinical challenge and is associated with chronic symptoms, functional impairment, and repeated exposure to pharmacological and non-pharmacological treatment strategies. Although the most used definition refers to insufficient response to at least two antidepressant trials of adequate dose and duration, there is no universally standardized definition with established predictive utility for treatment selection or clinical outcomes. Proposed definitions vary with respect to the number, duration, dose adequacy, and pharmacological class of failed treatments, as well as whether psychotherapy, augmentation strategies, electroconvulsive therapy (ECT), or adherence are considered. This heterogeneity complicates comparisons across studies and should be considered when interpreting findings in TRD cohorts (1). We applied the European Medicines Agency (EMA) criteria in our study (see Methods section and Participants section) (2).

Ketogenic interventions have been discussed as potentially relevant across mood disorders and severe psychiatric illness more broadly. A narrative review of ketogenic therapy for major depressive disorder summarized preliminary clinical and neurobiological evidence suggesting that nutritional ketosis may influence depressive symptoms by affecting cerebral energy metabolism, mitochondrial function, oxidative stress, inflammation, the gamma–aminobutyric acid/glutamate balance, neurotrophic signaling, and the gut-brain axis (3). A retrospective analysis of 31 inpatients with refractory mental illness, including major depressive disorder, bipolar disorder, and schizoaffective disorder, reported that adjunctive ketogenic dietary treatment in a semi-controlled inpatient setting was feasible, well tolerated, and associated with improvements in depression and psychosis symptoms as well as metabolic health markers (4). However, these data were limited by retrospective designs and diagnostic heterogeneity. Thus, while early findings supported the rationale for ketogenic metabolic therapy in mood disorders, clinical evidence specifically examining ketogenic diet interventions in treatment-resistant depression remained very limited when recruitment for the present study began on 1 November 2023.

The present study was designed as a pilot trial to examine whether a 6-week KD would be feasible in patients with TRD in a real-world psychiatric setting, and whether adherence, safety, and a preliminary antidepressant signal would justify larger future studies. The primary aim of the present report is therefore descriptive: to characterize recruitment, implementation, achievement of nutritional ketosis, tolerability, and individual clinical trajectories in a prospective single-arm cohort.

## 2 Methods

### 2.1 Study design

This was a prospective, single-arm, open-label 6-week pilot trial conducted at the Medical University of Vienna, Department of Psychiatry and Psychotherapy, Division of General Psychiatry. Recruitment took place between 1 November 2023 and 30 September 2025 in both inpatient and outpatient settings. The study received prior approval from the Ethics Committee of the Medical University of Vienna (EK 1882/2020). The study protocol was not prospectively registered in a public trial registry and was not made publicly available.

### 2.2 Participants

Eligible participants were men and women aged 18–65 years receiving treatment at the outpatient clinic or inpatient wards of the Division of General Psychiatry. The key inclusion criterion was TRD during a current moderate (ICD-10: F33.1) or severe (ICD-10: F33.2) depressive episode with MADRS ≥20. For TRD, we applied the European Medicines Agency (EMA) criteria (2). Thus, TRD was defined as an insufficient response to antidepressant treatment from at least two different pharmacological classes over at least 4 weeks. Eligibility also required stable psychopharmacological treatment for at least 1 week before starting the KD and a body mass index (BMI) of 19–35 kg/m^2^. Major exclusion criteria included psychotic depression, bipolar disorder, schizophrenia spectrum disorders, substance use disorders (excluding nicotine), acute suicidality, severe metabolic or malignant disease, insulin-dependent diabetes or treatment with sulfonylureas, glinides, or SGLT-2 inhibitors, nephrolithiasis, orthostatic dysregulation, inflammatory bowel disease, celiac disease, pregnancy, clinical circumstances suggesting that dietary adherence would be unrealistic, and the use of antibiotics within the previous 3 months.

### 2.3 Intervention

The intervention consisted solely of a 6-week KD. Existing psychopharmacological treatment was not changed during this period. The intervention could be implemented in an inpatient, outpatient, or mixed inpatient/outpatient setting. The diet focused on fatty meat, fatty fish, eggs, full-fat cheese, cream, olive oil, butter, berries, vegetables, and nuts. A 6-week ketogenic meal plan was developed by a dietitian. For inpatients, ketogenic meals were provided by an external kitchen according to this plan. A representative day provided approximately 2,000 kcal, 171 g of fat, 96 g of protein, and 21 g of carbohydrates. Outpatients either collected ketogenic meals from the same external kitchen or prepared their meals at home. They received the meal plan as a template, an individualized list of permitted foods, and dietary instructions to limit carbohydrate intake to a maximum of 30 g/day and protein intake to a maximum of 100 g/day. Weight loss was not presented as a treatment target; instead, patients were instructed to include sufficient fat with each meal and eat to satiety.

### 2.4 Adherence and nutritional ketosis monitoring

Adherence was monitored by daily fasting capillary blood ß-hydroxybutyrate (BHB) measurements obtained by finger prick using the FreeStyle Precision Neo (Abbott) and ketone test strips. For the present report, the first day of nutritional ketosis was defined as the first day with a BHB level of >0.5 mmol/L. Mean BHB levels across the documented study period were calculated for each patient.

### 2.5 Clinical and laboratory assessments

MADRS was obtained at baseline and after 2, 4, and 6 weeks. The Beck Depression Inventory-II (BDI-II) was administered at baseline and after 6 weeks. Ratings at baseline and at week 6 were conducted by trained clinical psychologists who were not involved in patient care. Routine laboratory investigations included complete blood count, blood chemistry, thyroid hormone levels, and HbA1c. Although serum cytokine, microbiome, and leaky gut analyses were part of the broader protocol framework, they were not analyzed in the present study due to the small sample size.

### 2.6 Outcomes

Because of the pilot nature of the study and the small sample size, outcomes were analyzed descriptively. The main domains of interest were feasibility of recruitment, retention, achievement of nutritional ketosis, tolerability, and individual clinical trajectories. Response was defined as a ≥ 50% reduction in the MADRS score from baseline. Remission was defined as a MADRS score of ≤10.

### 2.7 Post-study structured patient interviews regarding the feasibility of the KD

After the study period, we conducted structured phone interviews with the available study participants to obtain exploratory feedback on motivations, barriers, and facilitators of their dietary adherence (or lack thereof). Six of the eight enrolled participants were reached. All interviewed patients were asked the same structured questions addressing their motivation for study participation, whether they had independently re-tried the KD after study completion, the perceived difficulty of carbohydrate restriction, deviations from the dietary protocol, difficulties in social situations, physical problems during the diet, and perceived changes in mood and physical performance.

### 2.8 Statistics

Given the small sample size and marked heterogeneity in completion status, only descriptive statistics are reported. Continuous variables are summarized as means. Individual patient trajectories are presented narratively.

## 3 Results

### 3.1 Recruitment and participant flow

A total of 13 patients met the eligibility criteria and were offered study participation. Five declined participation: one preferred treatment with ECT, two preferred inpatient treatment with esketamine nasal spray, one because of an ongoing house move and lack of time for dietary change, and one felt a dietary change was infeasible. Eight patients were enrolled.

Of the eight enrolled patients, four completed the full 6-week intervention (case report forms [CRFs] 1, 3, 4, and 7). One patient (CRF 5) discontinued after week 2. One patient (CRF 2) was excluded after bipolar disorder and substance use were identified shortly after inclusion. Two patients did not achieve nutritional ketosis and discontinued early (CRFs 6 and 8).

### 3.2 Baseline characteristics

The eight enrolled participants comprised four men and four women. Four were recruited from the outpatient clinic and four from the inpatient wards. Depression severity at baseline was high, with MADRS scores ranging from 24 to 36 among the completers and from 30 to 31 among the non-completers. Table 1 shows the baseline demographic and clinical characteristics of the enrolled patients.

**Table 1:** Baseline demographic and clinical characteristics.

CRF	Sex	Age, years	Setting at entry	Primary psychiatric diagnosis	Somatic comorbidities	Baseline MADRS	Baseline BDI-II
1	Male	64	Inpatient	F33.1	Hyperlipidemia	24	33
2	Female	30	Inpatient	Initially, F33.2; later bipolar depression/substance use	None	31	36
3	Male	27	Outpatient	F33.2	Hypertension, hyperlipidemia, fatty liver, obesity	31	41
4	Female	45	Outpatient	F33.2	None	30	42
5	Female	58	Inpatient	F33.2	Hypertension	31	41
6	Male	34	Outpatient	F33.2	none	31	45
7	Male	57	Outpatient	F33.2	Hypertension, hyperlipidemia	36	38
8	Female	51	Outpatient	F33.2	Hypertension, hyperlipidemia	30	28

MADRS, Montgomery-Åsberg Depression Rating Scale; BDI-II, Beck Depression Inventory-II; ICD-10, International Classification of Diseases, 10th Revision; F33.1, recurrent depressive disorder, current moderate episode; F33.2, recurrent depressive disorder, current severe episode without psychotic symptoms.

### 3.3 Nutritional ketosis monitoring

Daily fasting capillary BHB measurements were used to monitor adherence. The first documented day of nutritional ketosis (a BHB level of >0.5 mmol/L) was day 3 for CRF1, day 2 for CRF 3, CRF 4, and CRF 5, and day 4 for CRF 7. Mean BHB over the documented study period was 1.31 mmol/L for CRF 1, 0.52 mmol/L for CRF 3, 3.09 mmol/L for CRF 4, 1,61 mmol/L for CRF 5, and 1.23 mmol/L for CRF 7. CRF 6 reached a BHB level of >0.5 mmol/L on only 2 days over a 2-week period, despite self-reported strict adherence. CRF 8 did not achieve nutritional ketosis during the 8-day period of home-based dietary implementation, with BHB values ranging from 0.1 to 0.3 mmol/L. Table 2 summarizes the individual nutritional ketosis and clinical trajectories during the 6-week study period.

**Table 2:** Individual nutritional ketosis and clinical trajectories during the 6-week study period.

CRF	First day with BHB >0.5 mmol/L	Mean BHB, mmol/L	MADRS baseline	MADRS week 2	MADRS week 4	MADRS week 6	MADRS response	MADRS remission	Notes
1	3	1.31	24	8	3	5	Yes	Yes	Rapid early improvement
2	n/a	n/a	31	n/a	n/a	n/a	n/a	n/a	Excluded after revised diagnosis
3	2	0.52	31	16	21	10	Yes	Yes	Transient fluctuation before endpoint remission
4	2	3.09	30	12	9	31	No	No	Early response not sustained
5	2	1.61	31	12	n/a	n/a	Yes, at week 2	No at week 2	Discontinued after week 2
6	n/a	n/a	31	31	n/a	n/a	No	No	Failed to achieve sustained nutritional ketosis
7	4	1.23	36	37	38	36	No	No	No clinical improvement despite sustained nutritional ketosis
8	n/a	0.20	30	30	n/a	n/a	No	No	Failed to achieve nutritional ketosis

BHB, ß-Hydroxybutyrate; MADRS, Montgomery-Åsberg Depression Rating Scale.

### 3.4 Case summaries

#### 3.4.1 CRF 1

CRF 1 was a 64-year-old man with recurrent depressive disorder (currently in a moderate episode) and hyperlipidemia. He was recruited from the inpatient clinic. Five months before enrolment, he had undergone intranasal esketamine treatment without a relevant clinical benefit. At study entry, his medication consisted of escitalopram 30 mg, Deanxit, one tablet daily (containing 0.5 mg flupentixole and 10 mg melitracen), and rosuvastatin 10 mg. Lorazepam 1 mg was available as needed for episodes of increased inner tension and was taken irregularly. Baseline routine laboratory test results were unremarkable.

At study entry, his main depressive symptoms were severe exhaustion, loss of drive, inner restlessness, diffuse anxiety, and anhedonia. His baseline MADRS score was 24, and his BDI-II score was 33. Nutritional ketosis was first documented on day 3. The mean BHB level over the documented study period was 1.31 mmol/L.

Clinical improvement was evident after 2 weeks, as the MADRS score decreased to 8, then further declined to 3 at week 4 and slightly increased to 5 at week 6. The BDI-II score decreased to 3 at week 6. The patient therefore met the criteria for both response and remission. Symptomatic improvement was particularly reflected in reduced exhaustion, restlessness, anxiety, and anhedonia. Because of the rapid improvement, he was discharged from inpatient care after 2 weeks and completed the remaining 4 weeks in the outpatient setting.

The ketogenic diet was generally well tolerated. Adverse effects included a bland taste in the mouth and bilateral nocturnal calf cramps during the first 2 weeks. Serum uric acid increased transiently from 6.8 to 9.4 mg/dL after 2 weeks and later declined without pharmacological intervention. ApoB remained stable, and thyroid hormone levels remained unchanged despite a mild increase in thyroid-stimulating hormone. At a follow-up visit 9 months after study completion, his MADRS score was 9 with unchanged psychopharmacological treatment, suggesting sustained remission, although he had followed the ketogenic diet only intermittently in 4-week periods rather than continuously.

#### 3.4.2 CRF 2

CRF 2 was a 30-year-old woman who was initially enrolled with a presumed diagnosis of recurrent depressive disorder (currently in a severe episode). She was recruited from the inpatient clinic.

At baseline, her MADRS score was 31, and her BDI-II score was 36. Her main depressive symptoms were consistent with a severe depressive episode, but shortly after inclusion, bipolar depression and clinically relevant substance use became evident, including withdrawal symptoms and substance use during leave from the ward. Because the revised diagnoses were incompatible with the study criteria, the patient was excluded after 3 days.

#### 3.4.3 CRF 3

CRF 3 was a 27-year-old man with recurrent depressive disorder (currently in a severe episode) and relevant somatic comorbidities, including obesity, dyslipidemia, hypertension, and fatty liver disease. He was recruited from the outpatient clinic. At study entry, medication consisted of venlafaxine 300 mg, bupropion 300 mg, tianeptine 60 mg, lamotrigine 250 mg, topiramate 50 mg, pregabalin 50 mg, methylphenidate 36 mg, amlodipine 10 mg, lisinopril 15 mg, bezafibrate 400 mg, folic acid 5 mg, omega-3 supplementation, and vitamin D supplementation. Baseline laboratory test results were notable for a triglyceride level of 308 mg/dL, an elevated total cholesterol level of 241 mg/dL, and an LDL cholesterol level of 146 mg/dL.

At study entry, his baseline MADRS score was 31, and his BDI-II score was 41. His main depressive symptoms included sadness, pessimistic thinking, inner tension, impaired concentration, reduced drive, and sleep disturbance. Nutritional ketosis was first documented on day 2. The mean BHB level over the documented study period was 0.52 mmol/L.

His MADRS score improved to 16 at week 2, worsened transiently to 21 at week 4, and improved again to 10 at week 6, meeting the criteria for both response and remission. The transient worsening at week 4 occurred after an approximately 10-day interruption of the ketogenic diet because of gastroscopy-related procedures, after which the patient resumed the intervention and improved again by week 6. Symptomatic improvement was particularly noted in sadness and pessimistic thinking, as well as inner tension, concentration, and drive. Sleep also improved during the study.

The ketogenic diet was generally well tolerated. Adverse effects were limited to transient diarrhea during the first few days. After 6 weeks, triglyceride and total cholesterol levels had decreased, while LDL cholesterol levels remained approximately unchanged. The patient reportedly continued the ketogenic diet for 4 weeks after study completion and initially felt well, but after returning to a high-carbohydrate diet during a family vacation, he described renewed worsening of drive, mood, and concentration.

#### 3.4.4 CRF 4

CRF 4 was a 45-year-old woman with recurrent depressive disorder (currently in a severe episode, with illness onset in adolescence). One year before study entry, she had shown a substantial response to an intranasal esketamine series, followed by maintenance treatment every 2 weeks. Despite ongoing maintenance treatment, her depressive symptoms worsened again. She was recruited from the outpatient clinic and completed the intervention entirely as an outpatient. At study entry, her medications included lamotrigine 50 mg and alprazolam 0.5 mg as needed, which were taken intermittently. In addition, she received regular intranasal esketamine maintenance treatment every 2 weeks. Baseline routine laboratory test results were unremarkable.

At study entry, her baseline MADRS score was 30, and her baseline BDI-II score was 42. Her main depressive symptoms included sadness, hopelessness, inner tension, appetite loss, impaired concentration, and reduced drive. Nutritional ketosis was first documented on day 2. The mean BHB level over the documented study period was 3.09 mmol/L, the highest among the completers.

Her MADRS score improved markedly to 12 at week 2 and 9 at week 4, but increased again to 31 at week 6. Early improvement was seen mainly in reduced sadness, hopelessness, inner tension, appetite loss, as well as improved concentration and drive. By the end of week 6, however, her depressive symptom severity had returned to baseline levels.

During the first 3 weeks, the ketogenic diet was well tolerated. From week 4 onward, she reported fatigue, orthostatic dizziness, calf cramps, dry mouth, and nausea without vomiting or diarrhea. Notably, these symptoms began after one of her routine biweekly intranasal esketamine maintenance treatments, although a causal relationship to esketamine, the ketogenic diet, or their combination cannot be inferred. These adverse effects improved only minimally with a sugar-free electrolyte supplement. Her LDL cholesterol level increased from 87 to 123 mg/dL, and her ApoB level increased from 75 to 102 mg/dL, while other routine laboratory parameters remained stable. She did not continue the ketogenic diet after study completion. At a follow-up visit approximately 3 months after study completion, her MADRS score was 35.

#### 3.4.5 CRF 5

CRF 5 was a 58-year-old woman with recurrent depressive disorder (currently in a severe episode) and hypertension. She was recruited from the inpatient unit after 8 weeks of hospitalization without sufficient clinical improvement. During her admission, she had undergone nine intranasal esketamine treatments with transient mood brightening on treatment days but no sustained benefit. At study entry, her medication consisted of bupropion 300 mg, prothipendyl 120 mg, aripiprazole 5 mg, pregabalin 400 mg, trazodone 50 mg, venlafaxine 225 mg, amlodipine 5 mg, folic acid supplementation, and omega-3 supplementation. Baseline routine laboratory tests were unremarkable.

At hospital admission, her MADRS score had been 41. At inclusion in the study, her MADRS score was 31, and her BDI-II score was 41. Her main depressive symptoms were sadness, loss of drive, anhedonia, exhaustion, social withdrawal, inner tension, self-reproach, concentration problems, insomnia, passive death wishes, and self-care deficits. The ketogenic diet was implemented on the ward using meals provided by the external kitchen. Nutritional ketosis was achieved on day 2. The mean BHB level over the documented study period was 1.61 mmol/L.

At week 2, her MADRS score improved to 12, and her BDI-II score improved to 12, with improvement seen mainly in reduced sadness, anhedonia, and inner tension, as well as improved appetite and drive.

The ketogenic diet was initially feasible on the ward. After 2 weeks, however, the patient wished to discontinue inpatient treatment and study participation. Financial concerns about continuing the diet at home were initially raised; after a meal pick-up system from the external kitchen was organized, she additionally reported nausea related to the fat-rich diet and subsequently withdrew from the study. No relevant pathological laboratory changes during the ketogenic diet were documented before discontinuation. Because she did not remain in the protocol beyond week 2, formal follow-up data within the study were not available.

#### 3.4.6 CRF 6

CRF 6 was a 34-year-old man with recurrent depressive disorder (currently in a severe episode), recruited in the outpatient setting after discharge from a 4-month inpatient psychiatric stay. He had a long history of highly treatment-resistant illness with prior intranasal esketamine treatment and ECT, neither of which had yielded a sustained benefit. At study entry, his medications included duloxetine 120 mg, mirtazapine 30 mg, lamotrigine 150 mg, aripiprazole 5 mg, lisdexamfetamine 50 mg, bisoprolol 2.5 mg, and tizanidine 8 mg. Baseline routine laboratory test results were unremarkable.

At study entry, his baseline MADRS score was 31, and his BDI-II score was 45. His main depressive symptoms were consistent with severe treatment-resistant depression, including persistent depressive mood and marked functional impairment. The patient implemented the ketogenic diet at home. Despite self-reported strict adherence, he did not achieve sustained nutritional ketosis. During the 2 weeks of dietary treatment, his BHB levels exceeded 0.5 mmol/L on only 2 days.

His MADRS score remained unchanged after 2 weeks, and no clinically relevant antidepressant effect was observed. The main tolerability issue was not a classical adverse effect but rather frustration related to the lack of nutritional ketosis and absence of clinical improvement. Follow-up laboratory test results after 2 weeks showed no major changes. He discontinued the intervention. Approximately 3 months after study completion, because of persistent severe depression, a vagus nerve stimulator was implanted.

#### 3.4.7 CRF 7

CRF 7 was a 57-year-old man with recurrent depressive disorder (currently in a severe episode), recruited in the outpatient clinic. His somatic comorbidities included hypertension and hyperlipidemia. At study entry, his medications consisted of venlafaxine 150 mg and olanzapine 10 mg. Alprazolam 0.5 mg was available as needed and was taken an average of approximately two tablets per week. Baseline routine laboratory test results were unremarkable apart from dyslipidemia.

At study entry, his baseline symptoms included sadness, anhedonia, rumination, guilt, inner tension, impaired concentration, reduced drive, and passive death wishes. His baseline MADRS score was 36, and his BDI-II score was 38. Nutritional ketosis was first documented on day 4. The mean BHB level over the documented study period was 1.23 mmol/L.

Despite achieving sustained nutritional ketosis, no relevant antidepressant effect was observed. MADRS was 37 at week 2, 38 at week 4, and 36 at week 6. His BDI-II score was 42 at week 6. The patient reported that the ketogenic diet may have even worsened his nervousness and hopelessness.

The KD was generally well tolerated, apart from initial constipation and dry mouth. Routine laboratory test results remained largely stable except for the LDL cholesterol level, which increased from 125 to 174 mg/dL. Two months after study completion, he required inpatient admission because of persistent severe depression.

#### 3.4.8 CRF 8

CRF 8 was a 51-year-old woman with recurrent depressive disorder (currently in a severe episode). She had previously received an intranasal esketamine series with a partial clinical response. At study entry, her medications consisted of escitalopram 20 mg, trazodone 200 mg, amlodipine 5 mg, valsartan 240 mg, hydrochlorothiazide 12.5 mg, rosuvastatin 10 mg, and ezetimibe 10 mg. Baseline laboratory test results were unremarkable.

At study entry, her baseline MADRS score was 30, and her BDI-II score was 28. Her main depressive symptoms included sadness, anhedonia, inner tension, concentration problems, and reduced drive. She implemented the ketogenic diet at home but did not achieve nutritional ketosis and discontinued the intervention after 8 days. Her daily BHB values were 0.1, 0.2, 0.2, 0.2, 0.2, 0.3, 0.3, and 0.1 mmol/L. Her MADRS score was unchanged after 8 days, and no clinically relevant antidepressant effect was observed. No specific adverse effects of the ketogenic diet were documented; the main issue was the failure to achieve nutritional ketosis despite apparently good adherence.

Two months after study discontinuation, she was admitted for an ECT series at our inpatient clinic and improved her MADRS score from 25 to 10 after 10 sessions.

### 3.5 Post-study patient feedback on dietary feasibility

Structured post-study telephone interviews were completed with six of the eight enrolled patients. The most frequent motivation for participation was the hope for symptom improvement after experiencing insufficient benefit from previous treatments. One patient also emphasized weight loss as a major motivation. Three patients did not attempt the KD again after study completion because they had experienced no benefit, had not achieved nutritional ketosis, or had found the diet unpleasant or impractical. In contrast, two patients reported repeated use of the KD after the study. CRF 1 reported continuing the KD for 6 weeks after study completion and resuming it again approximately 9 months after study completion during a period of worsening mood, with perceived improvement in mood and drive. CRF 3 reported repeated use after study completion, mainly because he experienced the KD as a comparatively sustainable weight-loss strategy and perceived improvements in motivation, concentration, fatigue, and mood.

Carbohydrate restriction was described as feasible but burdensome by several patients. Bread, pasta, potatoes, and other starchy foods were more frequently missed than sweets or soft drinks. Perceived early mental or physical improvement appeared to facilitate adherence in some patients. Most interviewed patients reported strict adherence during the study period, although CRF 7 reported occasional deviations. Social situations were variably experienced: several patients reported few or no social barriers, partly because of limited social activity during severe depression or support from family and friends, whereas CRF 3 described workplace meals with colleagues as challenging.

Physical symptoms reported in the post-study interviews were consistent with the tolerability findings during the trial and included transient gastrointestinal symptoms, constipation, a bland taste in the mouth, fatigue, headache, dizziness, and weakness. Perceived changes in mood and performance were heterogeneous. CRF 1 and CRF 3 reported subjective improvement, particularly in improved mood, drive, and concentration, and reduced fatigue. CRF 4 reported increased energy during the first 3 weeks but a worsening of symptoms during the second half of the intervention. CRF 6, CRF 7, and CRF 8 did not report any relevant improvement in mood; CRF 7 additionally perceived reduced physical performance, particularly during exercise.

## 4 Discussion

This prospective, single-arm, open-label pilot study suggests that a ketogenic diet can be implemented in a subset of patients with TRD. It may be associated with rapid clinical improvement in some individuals. Rapid symptomatic improvement was observed in several patients, particularly CRF 1, CRF 3, and CRF 4 during the first 4 weeks, and CRF 5 during the first 2 weeks. However, this improvement was not sustained or fully evaluable in all cases. At the same time, the study highlights substantial challenges in recruitment, maintenance, and the heterogeneity of both nutritional ketosis achievement and antidepressant response.

First, recruitment proved more difficult than expected. Although the original plan had been to include 20 patients, only 8 were enrolled during the recruitment period. Restricting the ketogenic diet to treatment-resistant, very ill patients imposed limitations on patient recruitment. Two factors likely played an important role. From 2020 onward, patients with TRD in our specialized outpatient service were increasingly treated with intranasal esketamine spray, in some cases successfully, thereby reducing the number of otherwise eligible ambulatory candidates. In the inpatient setting, ECT was a well-established and often expected treatment option; some patients were admitted with a prior expectation that they would receive ECT during hospitalization. In this clinical context, participation in a dietary intervention may have appeared less attractive than established biological treatments that offered more immediate therapeutic credibility and less personal effort than changing eating habits. The post-study interviews provide additional context for these feasibility findings. Patients generally entered the study because they hoped for symptom reduction after multiple unsuccessful previous treatments, suggesting that a willingness to attempt a demanding dietary intervention may be highest in patients with a substantial unmet therapeutic need. However, the interviews also indicate that adherence was not determined solely by motivation. The practical burden, the taste and acceptability of the KD, missing starchy staple foods, social eating contexts, work demands, fatigue, and the absence of early perceived benefit all appeared to be relevant factors. Conversely, early subjective improvement, family support, and the ability to explain the diet in social settings appeared to facilitate adherence. The observation that patients with perceived benefit were more likely to retry the KD after study completion, whereas those who experienced no benefit or failed to achieve nutritional ketosis did not, is clinically plausible and highlights the importance of early metabolic feedback and perceived symptom change for sustained implementation.

In addition, the use of antibiotics within the preceding 3 months also prevented otherwise suitable patients from being included in the screening. We defined this as an exclusion criterion because gut microbiome analyses had initially been planned as part of the study protocol. Recent antibiotic exposure was considered a potential confounder, as antibiotics can alter gut microbiome composition and could therefore have biased the microbiome-related analyses (5). However, because of the small final sample size, the gut microbiome analyses were ultimately not performed.

Second, the achievement and depth of nutritional ketosis were not uniform. Among the participants who achieved nutritional ketosis, the mean BHB concentrations over the documented study period varied substantially, ranging from 0.52 mmol/L in CRF 3 to 3.09 mmol/L in CRF 4. This variability is clinically relevant because a simple dichotomous classification of participants as “ketotic” or “non-ketotic” may obscure potentially important differences in metabolic exposure. For example, CRF 3 met the criteria for nutritional ketosis but had a comparatively low mean BHB concentration and an interrupted dietary course, whereas CRF 4 reached the highest mean BHB concentration among the completers but showed only transient clinical improvement. CRF 5 achieved a mean BHB level of 1.61 mmol/L and showed marked improvement after 2 weeks but discontinued the intervention early, while CRF 7 achieved sustained nutritional ketosis with a mean BHB of 1.23 mmol/L but showed no antidepressant response. In contrast, two patients failed to achieve nutritional ketosis despite apparently good adherence in the outpatient setting. CRF 6 reached BHB concentrations >0.5 mmol/L on only 2 days during the 2 weeks despite self-reported strict adherence, and CRF 8 did not achieve nutritional ketosis during the 8 days of home-based dietary implementation, with BHB values ranging from 0.1 to 0.3 mmol/L. This may have several explanations. One possibility is that self-reported adherence overestimates actual dietary precision, especially in home-based implementation without full control of food intake. Even small hidden carbohydrate exposures may be sufficient to prevent nutritional ketosis. Additional factors may include differences in total caloric intake, protein intake, insulin resistance, medication effects, physical activity, sleep, and stress physiology.

Third, the clinical response was heterogeneous even among those who achieved nutritional ketosis. CRF 1 and CRF 3 showed marked improvement at week 6. CRF 4 showed a striking early improvement and later loss of benefit by week 6, possibly related to worsening tolerability with discontinuation immediately after the protocol. CRF 5 demonstrated promising early improvement, but she discontinued the intervention after the second week, mainly for organizational reasons. CRF 7, in contrast, achieved nutritional ketosis but did not show clinical improvement. This dissociation suggests that nutritional ketosis alone may not be sufficient for an antidepressant benefit and that any effect of ketogenic interventions in patients with TRD is likely moderated by clinical subtype, illness chronicity, metabolic status, concurrent treatments, or other unmeasured factors.

The present findings should be interpreted considering the literature available at the time the study was initiated. When recruitment began in November 2023, there was essentially no clinical trial evidence specifically addressing ketogenic diet interventions in TRD. The pilot nature of the present work was therefore justified, particularly with respect to adherence, feasibility, and the generation of preliminary effect-size estimates for later studies. Since then, randomized controlled evidence in TRD has emerged, suggesting a modest antidepressant benefit of the KD compared with a well-matched control diet (6).

This study has several limitations. The sample was very small and clinically heterogeneous. The lack of a control group and the use of an open-label design limited causal inferences and may have increased susceptibility to placebo effects. Participants who achieved nutritional ketosis and completed the intervention might represent a more motivated, adherent, or metabolically responsive subgroup than the broader TRD population, a point that should be acknowledged given the study’s focus on feasibility. The analytic dataset was affected by early exclusions and discontinuations. Various concurrent psychopharmacological treatments represent an important potential confounder in this study. Although preexisting psychopharmacological medication had to be stable at least 1 week before study entry and was not changed during the 6-week ketogenic intervention, ongoing treatment exposure may still have influenced clinical trajectories. This is particularly relevant for patients with recent or ongoing intranasal esketamine treatment. CRF 4 had previously responded to an intranasal esketamine series and continued biweekly esketamine maintenance treatment during the ketogenic diet. Therefore, her early symptomatic improvement and subsequent worsening could be attributed to any of the following: the ketogenic diet, esketamine maintenance with extended intervals, or the natural fluctuation of severe recurrent depression. In the absence of a control group, these concurrent medications limit the causal interpretation of the antidepressant effects observed during the ketogenic intervention.

Although the ketogenic dietary intervention was based on a dietitian-developed 6-week meal plan and predefined carbohydrate and protein limits, dietary implementation was not fully centralized across all participants. Inpatients received prepared ketogenic meals, whereas outpatients used the meal plan as a template and implemented the ketogenic diet through meal pick-up or home preparation. Thus, the actual dietary composition varied between participants in the outpatient setting. This may have contributed to variability in adherence, the depth of nutritional ketosis, tolerability, metabolic responses, and clinical outcomes. Future controlled trials should use more detailed dietary documentation and, where feasible, more standardized meal provision across settings. Microbiome and leaky gut analyses, although originally envisioned in the wider protocol framework, were not performed due to the small sample size.

The post-study interviews were exploratory and retrospective; only six of the eight patients could be reached, and their responses were summarized descriptively without formal qualitative coding. Recall bias cannot be excluded. We chose a 6-week observation period without having reliable evidence regarding the latency period or the maintenance of ketogenic effects.

However, the study also has several strengths. It was conducted prospectively in a real-world academic psychiatric setting and included both inpatient and outpatient implementation. Daily ketone monitoring provided a biologically meaningful measure of adherence. The case summaries illustrate the diversity of clinical trajectories in TRD and may be useful for hypothesis generation regarding candidate subgroups, adverse effects, and practical barriers.

In conclusion, this pilot study suggests that a ketogenic diet may be feasible and clinically promising for some patients with TRD who are prepared for the complex dietary change and BHB measurements. During this process, they need access to regular medical consultations, at least during the first 6 weeks. Recruitment barriers, early discontinuations, variable tolerability, and the inconsistent achievement of nutritional ketosis remain major challenges. Larger controlled studies are needed to identify which patients are most likely to benefit and under which implementation conditions ketogenic interventions can be sustained. Biological markers that predict a good antidepressant response to a ketogenic diet are unknown. Future investigations of immune status, microbiome, and intestinal barrier permeability could provide further insights.

## Data Availability

The original contributions presented in the study are included in the article/supplementary material, further inquiries can be directed to the corresponding author.
